# Electrophysiological correlates of in vivo and virtual reality exposure therapy in spider phobia

**DOI:** 10.1111/psyp.14117

**Published:** 2022-06-10

**Authors:** Stefan Wiens, Rasmus Eklund, Malina Szychowska, Alexander Miloff, Danielle Cosme, Stephen Pierzchajlo, Per Carlbring

**Affiliations:** ^1^ Department of Psychology Stockholm University Stockholm Sweden; ^2^ Annenberg School for Communication University of Pennsylvania Philadelphia Pennsylvania USA

**Keywords:** EEG, ERP, psychopathology, specific phobia, therapy effects, virtual reality

## Abstract

Specific phobia can be treated successfully with exposure therapy. Although exposure therapy has strong effects on self‐reported ratings and behavioral avoidance, effects on measures derived from electroencephalography (EEG) are scant and unclear. To fill this gap, spider‐phobic individuals received either in‐vivo or virtual reality exposure treatment. Patients were tested twice (one week before and after treatment), and control subjects once. In each session, EEG was recorded to spider pictures as well as other positive, negative, and neutral pictures. During EEG recording, participants performed a simple detection task while task‐irrelevant pictures were shown in the background. The task was used to reduce potential confounding effects from shifts of attention. After the task, subjects were shown the pictures again and rated each in terms of their emotional reaction (arousal and pleasantness). The results showed that before treatment, patients rated spiders as more negative than did control subjects. Patients also showed elevated early posterior negativity (EPN) and late positive potential (LPP) to spiders. After treatment, the negative emotional ratings of spiders were substantially reduced. Critically, Bayesian analyses suggested that EPN and LPP were unaffected by treatment and that the treatment groups did not differ in their responses (EPN, LPP, and ratings). These findings suggest that the effects of in vivo and virtual reality exposure therapy are similar and that the initial stages of motivated attention (EPN and LPP) are unaffected by treatment.

## Abstract 2

Although specific phobia is treated successfully with exposure therapy, effects on EEG measures are scant and unclear. Spider‐phobic individuals received either in‐vivo or virtual reality exposure treatment. One week after therapy, emotion ratings to spiders were reduced whereas EPN and LPP were unaffected. Effects did not vary by treatment. Results suggest that initial stages of motivated attention are unaffected by treatment.

## 1 INTRODUCTION

Specific phobia is a debilitating fear of an object or situation and one of the most common psychological disorders (Wardenaar et al., 2017). The treatment of choice is exposure therapy because it is highly effective on self‐reported fear and behavioral avoidance, particularly so for in vivo exposure therapy (IVET) involving real‐life exposure to feared stimuli or situations (Wolitzky‐Taylor et al., 2008).

One‐session treatment (OST) is a modern implementation of IVET and consists of a single 3‐hr session (rather than multiple sessions), and includes a cognitive aspect to identify and target catastrophic fears (Davis et al., 2012). Fear of spiders, a frequently reported specific phobia (Oosterink et al., 2009), is efficiently treated with OST‐type IVET and results in decreased self‐reported fear (Zlomke & Davis, 2008) and behavioral avoidance (Andersson et al., 2009).

In recent years, virtual reality exposure therapy (VRET) has been developed for the treatment of spider phobia and other specific phobias (Carl et al., 2019; Maples‐Keller et al., 2017). Because VRET does not require a patient to interact with the live stimuli, it reduces reluctance to seek treatment (Garcia‐Palacios et al., 2007). Also, VRET does not require that live animals and insects are collected and stored (Reuterskiöld & Öst, 2012). VRET for spider phobia has shown good efficacy in randomized‐controlled trials (Garcia‐Palacios et al., 2002; Hoffman et al., 2003; Michaliszyn et al., 2010; Miloff et al., 2019; Minns et al., 2018; St‐Jacques et al., 2010). In the most recent trial (Miloff et al., 2019), several authors of the present study randomly assigned 100 spider phobia patients to either IVET (OST type) or VRET (with low‐cost hardware and an automated treatment format). Results showed that both therapies strongly reduced self‐reported fear and avoidance behavior.

However, there is scant evidence on the effects of exposure therapy on electroencephalography (EEG) measures in spider phobia (Bernhardsson et al., 2016; Leutgeb et al., 2009, 2012). In these IVET studies, EEG measures were interpreted in the context of motivational theories of emotion (Lang et al., 1997, 1998; Lang & Bradley, 2010). From this theoretical perspective, emotions are grounded in motivational systems that are either appetitive (related to approach) or defensive (related to avoidance). In studies with humans, people are typically presented with emotional and neutral pictures while various psychophysiological measures are recorded. The pictures are also rated on valence (from unpleasant to neutral to pleasant) and arousal (low to high). According to motivational theories of emotion, the direction of the motivation (appetitive or defensive) is represented by valence ratings, and the strength (low to high) is represented by arousal ratings. Relative to neutral pictures, pleasant, and unpleasant pictures are more motivating and thus capture attention more strongly. In support, when participants view arousing pictures (pleasant or unpleasant) rather than neutral pictures, they view the pictures longer (if participants themselves can control picture duration), rate them as more interesting, and show larger skin conductance responses (an index of sympathetic nervous system activation). Furthermore, when an acoustic startle probe is presented during picture viewing, the P3 to the probe is reduced more to arousing than neutral pictures, consistent with the idea that participants respond less to the startle probe because they attend more to arousing than neutral pictures (Schupp et al., 1997, 2004).

Among measures derived from EEG to emotional pictures, the early posterior negativity (EPN) and the late positive potential (LPP) have been observed most consistently (Cuthbert et al., 2000; Hajcak et al., 2011; Junghöfer et al., 2001; Olofsson et al., 2008; Schupp et al., 2006; Wiens & Syrjänen, 2013). They are obtained by subtracting amplitudes to neutral pictures from amplitudes to arousing (pleasant or unpleasant) pictures. Because EPN and LPP increase with motivational strength, EPN and LPP are observed in response to arousing pictures irrespective of valence (pleasant or unpleasant). The EPN is apparent as lateral occipital negativity between about 150 and 300 ms after stimulus onset, and the LPP is apparent as a central‐parietal positivity that starts after about 300 ms. Both EPN and LPP reflect attentional processes. In support, research that used fMRI to localize potential sources of EPN and LPP suggests regions involved in attention; EPN is generated mainly in lateral occipital regions (Junghöfer et al., 2006; Sabatinelli et al., 2013), and LPP is generated by a network of areas involving high‐order visual cortices and prefrontal cortex (Liu et al., 2012; Sabatinelli et al., 2005, 2006, 2009). Taken together, research supports the idea that EPN and LPP index effects of emotion on attention. In general, the effects of emotion on attention are referred to as motivated attention (Lang et al., 1997, 1998; Lang & Bradley, 2010), natural selective attention (Bradley, 2009), or emotional attention (Pourtois et al., 2013; Vuilleumier, 2005).

In specific phobias of spiders and snakes, EPN and LPP are enhanced to the feared pictures compared to neutral pictures. Although most studies focused on the LPP (Kolassa et al., 2005; Leutgeb et al., 2010; Michalowski et al., 2009; Miltner et al., 2005; Mühlberger et al., 2006; Norberg et al., 2010; Norberg & Wiens, 2013; Rosenbaum et al., 2020; Scharmüller et al., 2011; Schienle et al., 2008), several studies also reported EPN (Michalowski et al., 2009; Norberg & Wiens, 2013; Van Strien et al., 2009). As EPN and LPP amplitudes are enhanced to a similar degree in response to feared pictures compared to other negative pictures with similar arousal ratings (Michalowski et al., 2009), these findings of enhanced EPN and LPP to spiders in spider phobia are consistent with motivational theories of emotion.

If EPN and LPP are indexes of motivated attention (Bradley, 2009; Hajcak & Foti, 2020; Lang et al., 1997), then a reasonable prediction is that EPN and LPP decrease after therapy. However, only a few EEG studies have examined the effects of IVET in spider phobia, and these studies focused on LPP (Bernhardsson et al., 2016; Leutgeb et al., 2009, 2012). In line with the prediction, Bernhardsson et al. (2016) reported that spider phobics showed that therapy reduced LPP, and eye movement data suggested no avoidance of the spider pictures before and after therapy.

Because this report is available only as an abstract, however, these findings are preliminary. The second study examined the effects of therapy on the LPP (600–1200 ms) in spider‐phobic girls during passive picture viewing (Leutgeb et al., 2012). Results suggested that LPP decreased after therapy (as explained in the supplementary material, Wiens & Eklund, 2022). The third study measured the LPP (800–1500 ms) in spider‐phobic adults during passive picture viewing (Leutgeb et al., 2009). Results suggested that LPP increased rather than decreased after therapy. Although Leutgeb et al. (2009) argued that this increase in LPP makes theoretical sense, the reasoning is not entirely convincing, as described in the supplementary material (Wiens & Eklund, 2022). Taken together, the results of previous studies (Bernhardsson et al., 2016; Leutgeb et al., 2009, 2012) do not resolve whether LPP changes after therapy, and whether potential changes are observed for the EPN as well.

Building on these preliminary findings, the main goal of the present study was built on these preliminary findings to examine the effects of exposure therapy (IVET, VRET) on EPN and LPP to spider pictures (vs. neutral pictures) in spider phobia. Subjects were shown spider pictures as well as neutral, negative, and positive pictures. Other picture categories aside from spiders and neutral pictures were included to have a study design similar to that of previous studies (Bernhardsson et al., 2016; Leutgeb et al., 2009, 2012; Michalowski et al., 2009). However, because responses to pictures other than spiders and neutral pictures (i.e., positive and negative pictures) are of secondary interest for the main goal, they are reported in the supplementary material (Wiens & Eklund, 2022). The first main analysis examined whether the combined treatment groups differed from the control group in their responses to spiders versus neutral pictures. The second main analysis included only the two treatment groups and compared both groups in their responses to spiders versus neutral pictures before and after treatment. EEG, as well as picture ratings, were recorded approximately 1 week before and after treatment. The patients were a subset of the sample in the treatment study that compared the effects of IVET and VRET in spider phobia (Miloff et al., 2019). Because EPN is apparent at the very back of the head (Hajcak et al., 2011), we recorded high‐density EEG (64 electrodes) with whole‐head coverage to measure EPN as well as LPP. To minimize eye movements and shifts in attention during EEG recordings, participants performed a detection task on a small fixation cross that was superimposed on each picture and blinked occasionally. Although the task was simple, the blinking of the fixation cross was subtle to ensure that participants looked straight at the fixation cross.

In line with motivational theories of emotion (Bradley, 2009; Lang et al., 1997; Lang & Bradley, 2010), if patients rate spiders as less negative and arousing after treatment, motivated attention to spiders would be lower and thus EPN and LPP to spiders (vs. neutral pictures) should be lower after than before treatment. Also, if IVET and VRET do not differ in their ratings of spiders before and after treatment, both groups should show similar effects on EPN and LPP.

## 2 METHOD

All supplementary material is available at a university repository (Wiens & Eklund, 2022). The repository includes the rating, performance, and preprocessed EEG data; and the scripts to run the experiment and analyze all data. EEG data were preprocessed with the MNE package in Python (Appelhoff et al., 2019; Gramfort et al., 2013, 2014; Pernet et al., 2019). The remaining analyses were conducted with *RStudio* (RStudio Team, 2020) in *R* (R core Team, 2016) using several packages (Auguie, 2017; Bolker & Robinson, 2021; Bürkner, 2017; Gabry & Mahr, 2021; Goodrich et al., 2020; Kay, 2021; Kuznetsova et al., 2017; Lüdecke, 2021; Lüdecke et al., 2021; Schlegel & Steenbergen, 2020; Wickham, 2020; Wickham et al., 2019; Zhu, 2021). The main document is a detailed *R*‐markdown script. Its output is saved as a .html file and includes the reported analyses together with additional analyses (as described in the Results section).

### 2.1 Participants

The patient sample was a subset of spider‐phobic individuals included in a treatment study (Miloff et al., 2019, *N* = 100) that was registered (Miloff et al., 2016). All patients met the criteria for spider phobia (American Psychiatric Association, 2013) according to a structured clinical interview (SCID‐I/P, First et al., 2002) adapted for DSM‐5. Patients required an in‐vivo behavioral avoidance score of 9 points or less (out of 12), be 18 years or older to be included, and could not have another serious ongoing mental disorder, suicidal ideation, substance abuse, or be in concurrent mental health treatment, either psychotherapy or psychotropic medication (except when the dosage was stable for at least 3 months). The study also required that patients have no visual impairments such as lack of stereoscopic vision that would impede the virtual reality experience, however, prescription lenses and eyeglasses were acceptable.

The patient sample was randomized into two treatment groups (Miloff et al., 2019, *N* = 100). The reference treatment was gold‐standard in‐vivo exposure therapy (IVET) in the form of a modern massed one‐session treatment (referred to as OST, Öst et al., 1991) and led by a trained and supervised psychotherapist. The other treatment was a gamified virtual reality exposure therapy (VRET; Miloff et al., 2016) composed of multiple levels of increasingly realistic spiders, game‐playing elements such as points and puzzles, and a virtual embodied therapist (Miloff et al., 2020) to help guide the patient, reinforce progress, and provide psychoeducation.

Because the amendment of the ethical approval to collect EEG data was delayed until a few months after the beginning of the randomized trial, not all patients could be invited. Also, many patients participated only in the second session because they had completed their first session before EEG data collection received ethical approval. This suggests that missingness can be treated as missing completely at random.

Because of excessive EEG artifacts (defined below), one subject had to be excluded. The final patient sample comprised 33 participants in the IVET group and 37 participants in the VRET group. Of the 33 participants in the IVET group, 14 were tested both pre and post treatment, 2 were tested only pretreatment, and 17 were tested only post treatment. Of the 37 participants in the VRET group, 19 were tested both pre and post treatment, 2 were tested only pretreatment, and 16 were tested only post treatment. Patients were tested approximately 1 week before and 1 week after treatment.

The demographics and preclinical measures for the final patient sample (*N* = 70) were comparable to those for the sample in the randomized trial (Miloff et al., 2019). The respective measures were as follows for the present sample versus the main sample: mean age = 35.2 (*SD* = 11.1) versus 34.1 (10.4); proportion women = 82.9% versus 83.0%; mean pretreatment scores on behavioral avoidance test (BAT) = 5.2 (2.6) versus 5.2 (2.6); mean pretreatment scores on fear of spiders questionnaire (FSQ) = 96.9 (14.8) versus 97.0 (14.6); mean pretreatment scores on spider phobia questionnaire (SPQ) = 22.4 (3.5) versus 22.6 (3.4).

The final patient sample improved from pretreatment to posttreatment on BAT, FSQ, and SPQ. Linear‐mixed models of the interaction between group (VRET and IVET) and session (pre and post) were conducted with varying intercepts for participants. With regard to the change from pre to post across groups: BAT scores increased, mean = 4.4, 95%CI [3.9, 5.0]; FSQ scores decreased, mean = −37.4, 95%CI [−42.3, −32.6]; and SPQ scores decreased, mean = −8.0, 95%CI [−9.3, −6.7]. Results suggested that IVET decreased more strongly than VRET on FSQ scores, mean of interaction = 17.3, 95%CI [7.6, 27.0]; and on SPQ scores, mean of interaction = 3.3, 95%CI [0.7, 5.9].

The control group comprised students from local universities who participated only in a single session. Participants were informed that they would be shown pictures (negative, positive, neutral, and spiders) while EEG was recorded. There were no explicit inclusion criteria. Of the 53 participants, one subject was excluded because of excessive EEG artifacts. The remaining 52 students were 30 women and 22 men. All except two reported to be right‐handed. The mean age was 25.9 (*SD* = 6.7).

### 2.2 Material

The pictures were spiders, negative, positive, and neutral. For each category, 30 pictures (and one example picture) were taken from the International Affective Picture System (IAPS, Bradley & Lang, 2017; Lang et al., 2008). The official IAPS codes are reported as supplementary material. Because spider pictures have a clear figure‐ground organization, and because EPN and LPP are affected by nonemotional features such as composition (Bradley et al., 2007; Löw et al., 2013; Nordström & Wiens, 2012; Wiens et al., 2011), pictures other than spiders were selected to have a similar composition as spiders. Negative pictures included threatening animals, injured humans and animals, and guns; positive pictures included cute animals, babies, and happy couples; neutral pictures included inanimate objects and neutral facial expressions. Critically, differences in picture composition do not confound the main variables (group and time), as controls and patients viewed similar pictures before and after therapy.

The experiment was programmed in Presentation (www.neurobs.com). The visual stimuli were shown on a ViewSonic P227f cathode ray tube monitor (www.viewsonic.com) at a viewing distance of 57 cm; thus, 1 cm corresponded to a 1° visual angle. The IAPS pictures were shown with a 4:3 ratio (i.e., 20.9 × 15.7 cm) at the center of the screen on a black background. At the center of the screen, a small rectangle (0.4 cm × 0.4 cm) was superimposed in gray (RGB levels = 100, 100, 100) and contained a fixation cross (i.e., a plus sign). The fixation cross was slightly darker (108, 108, 108). However, because six subjects had difficulties in seeing the fixation cross, the contrast was increased for these subjects (maximum RGB levels = 130, 130, 130).

### 2.3 Procedure

After providing informed consent, participants were shown example pictures similar to those used during the actual experiment. The experiment comprised two tasks in a fixed order: detection task and picture rating task. EEG was recorded only during the detection task. A chin rest was used during both tasks to keep viewing distance constant.

#### 2.3.1 Detection task

Subjects were instructed to detect the blinking of the fixation cross (i.e., its disappearance) in the center of the computer screen while ignoring pictures presented in the background. A small, gray rectangle was shown at fixation throughout the task, and a fixation cross in a slightly lighter gray was superimposed within the rectangle. Subjects had to push the space key on a keyboard as soon as they detected that the fixation cross disappeared briefly (i.e., the luminance of the fixation cross changed to that of the rectangle for 100 ms).

Subjects were instructed to focus on the fixation cross during the entire task, to ignore the pictures that were shown in the background (centered at fixation), and to avoid blinking while pictures were shown (to minimize eye blink confounds on the EEG). Instructions were given verbally and also shown on the computer screen before the task started. To practice the task, subjects were familiarized with 30 trials that used the example pictures.

The task comprised 200 trials. Each trial consisted of a 1500‐ms picture followed by a blank interval that lasted randomly between 500 and 700 ms. The fixation cross blinked on 20% of the trials (i.e., 40 of 200 trials). These target trials occurred equally often for each picture category (i.e., 10 for each category). In target trials, the onset of the blink occurred between 300 and 1200 ms (in steps of 100 ms) after picture onset. These 10 possible onsets were used once in random order for each picture category. Trial order was pseudo‐randomized as follows: One of five consecutive trials was a target trial; thus, only two target trials could occur in a row. For a set of four targets, each of the four picture categories was used once (in random order). For a set of 12 nontargets, each picture category was used three times, and all nontargets were randomized with the restriction that no more than three nontargets in a row could be of the same category.

There were 30 pictures in each of the four picture categories because we planned to test each participant in three sessions with 10 unique pictures per category and session. Thus, for each participant, the 30 pictures in each category were divided randomly into three sets of 10 pictures (picture lists are reported as supplementary material). However, because we did not have the resources to collect data in a follow‐up session (i.e., session 3), different subsets of pictures were used for individual participants and sessions. Notably, varying the stimuli randomly across participants actually increases generalizability beyond a specific set of stimuli (Westfall et al., 2017).

In the task, each individual picture was used five times. For each picture category, the order of the pictures was randomized with the restriction that each unique picture from each picture category was used once before being used again (thus, 10 unique pictures × 5 repetitions × 4 categories = 200 trials).

Note that before the actual study, a pilot study (*n* = 9, student volunteers) was used to show that with the current stimulus parameters, participants would perform poorly in detecting the blinking of the fixation cross unless they focused on it. Results of the pilot study supported this conclusion (see supplementary material).

After the detection task, participants filled in a questionnaire about their experience of the task. Subjects rated how much they looked at the fixation cross, how easy it was to see the flash, how distracting the spider pictures were, how distracting the other pictures were, and how easy the task was in general. These questions had 9‐point scales with different anchors. In an open format, subjects could describe what (if anything) was particularly difficult about the task.

#### 2.3.2 Picture rating task

The task used the same 40 pictures (10 from each picture category) as in the detection task. On each trial, a picture was shown for 1500 ms. As in the detection task, a rectangle and a fixation cross were superimposed on the picture. After the picture, subjects had unlimited time to rate their feeling about the picture. The graphic depictions of the original self‐assessment manikin (SAM) rating scales of pleasure and arousal (Bradley & Lang, 1994) were shown on the screen in separate rows (pleasure icons above arousal icons). Nine gray circles were overlaid on the 9 levels of each rating scale (on and between the icons). For each trial, a small black ring was shown on top of the gray circle in the middle of the pleasure scale. As subjects moved the computer mouse horizontally, the ring moved to one of the 9 positions on the pleasure scale. Subjects chose a pleasure level by pressing the left mouse button. Then, the ring appeared in the middle of the arousal scale, and subjects used the computer mouse to rate arousal. For each session and subject, the direction of both scales together was randomly determined (i.e., pleasantness and high arousal to either left or right). After the ratings, the next picture was shown after 700 ms. Picture order was pseudo‐random with the restriction that each picture category was shown once in four trials. At the beginning of the task, subjects practiced this task by rating the four practice pictures.

### 2.4 EEG recording and preprocessing

The data from 64 standard positions (10–20 system) were recorded with an Active Two BioSemi system (BioSemi, Amsterdam, Netherlands). Data were sampled at 512 Hz and filtered with a hardware low‐pass filter at 208 Hz. No high‐pass filter was used. The analyses of the behavioral data during the detection task included all trials, whereas in the EEG data analyses, some trials were excluded (see below).

After high‐pass filtering of the continuous EEG data with a 0.1‐Hz Butterworth 4th degree two‐pass filter, all electrodes were re‐referenced to the average of all electrodes. Noisy electrodes were interpolated (spherical spline interpolation) from neighboring electrodes. The number of interpolated channels per recording were *M* = 0.96 (*SD* = 1.00) for the control group and *M* = 1.25 (*SD* = 1.27) for the experimental group. The independent component analysis *FastICA* (Hyvärinen & Oja, 2000), as implemented in MNE‐python, was used to correct eye blink artifacts. Components with the highest explained variance were visually inspected for the typical eye blink topography (Campos Viola et al., 2009). The number of components removed per recording were *M* = 1.08 (*SD* = 0.62) for the control group and *M* = 1.07 (*SD* = 0.44) for the experimental group. Epochs were extracted from −100 ms before picture onset to 1000 ms after picture onset. Each epoch was baseline corrected to the mean of the 100‐ms interval before tone onset (−100 to 0 ms).

For each recording, maximum amplitude ranges were extracted for individual epochs, and the distribution of these amplitude ranges was visually inspected. Individual trials that were apparent outliers were excluded. The exclusion thresholds were set for each individual and session because subjects showed substantial variability in these amplitude ranges. To reduce bias (Keil et al., 2014), this inspection was blind to the condition (i.e., picture category). Although inspection was not blind to patient status, only a negligible number of trials were removed (1.7% of all trials). However, later data‐driven detection of outliers was completely blind to patient status and condition (see below).

On the basis of visual inspection of the difference waves (spider minus neutral), the EPN was defined between 180 and 280 ms, and the LPP was defined between 300 and 700 ms after picture onset. For this interval and each picture category, mean amplitudes were extracted from P9, P7, PO7, P10, P8, and PO8 electrodes for the EPN‐relevant interval and C1, Cz, C2, CP1, CPz, CP2, P1, Pz, and P2 electrodes for the LPP‐relevant interval. Although this data‐driven approach increases the risk of false positives (Kriegeskorte et al., 2009; Makin & Orban de Xivry, 2019), we could not avoid it because we did not preregister the study before starting data analyses (Nosek et al., 2018). Thus, the results reported below are exploratory and cannot be considered confirmatory (Nosek et al., 2018). However, we argue that the choice of intervals and electrodes is a reasonable compromise between representing the data in the present study and avoiding overfitting. For example, overfitting would be a risk if odd electrodes are selected that may show effects in the present data but are unlikely to do so in other datasets. We note that results suggested that optimal measurement of the EPN would have required lower electrode positions, but these were not available with the head coverage of the EEG cap.

Mean amplitudes for the EPN‐relevant interval and LPP‐relevant interval were further screened for outliers in *R*. A violin plot of mean amplitudes across all trials and subjects (*N* = 31,000) suggested that any remaining outliers could be removed with a threshold of ±25 μV. A mean of 98.1% trials (min = 73.5%) were retained per subject and session.

### 2.5 Analyses of detection and rating task data

A series of linear mixed‐effects regression models were estimated on trial‐level data during the rating and detection tasks using a Bayesian approach. The data were not centered or standardized. Bayesian models were estimated using *brms* (Bürkner, 2017). In all Bayesian models, vague priors were used for intercepts and slopes (i.e., normal distribution with *M* = 0 and *SD* = 4).

The advantage of mixed‐effects models is that one can use all available data and is not limited only to patients with data in both sessions. That is, a primary strength of using multilevel modeling to assess change pre to post is that subjects with only one measurement can be included and listwise deletion need not be used. Indeed, it is critical to include all available data because otherwise power would be reduced and the estimated treatment effects would be biased (Matta et al., 2018).

Emotion ratings of the participants were modeled via a linear mixed‐effects ordinal regression. The response variable was modeled as ordinal to account for the fact that because rating scales contain psychological distances between response options (as opposed to objective distances), the boundary between response options cannot be assumed to be equidistant. For the EEG data, however, regular linear mixed‐effects models were used instead.

The multilevel models formally tested whether each picture category differed from the neutral condition. That is, we specified neutral pictures as the reference condition and, therefore, the parameter estimates represent the mean difference between neutral pictures and the images in each of the other picture categories. The results reported below focus on the difference between spiders and neutral pictures; however, the supplement contains comparisons for other picture categories (i.e., positive and negative vs. neutral).

For the main regression models of the rating and ERP data, the posterior distribution of the coefficients was estimated via Hamiltonian Markov Chain Monte Carlo (MCMC) simulations. We thus report the coefficients with their corresponding 95% highest‐density intervals (but refer to them as 95%CIs). Bayes Factor (BF) equivalents were estimated using a bridge sampling algorithm. It was chosen over conventional BF estimates as the output of mixed‐effect regression models is used to estimate the posterior distribution's normalizing constant and to compare it to the constant of a null model. Thus, the BF is the ratio of denominators from two Bayes Theorem equations. Although the BF is a continuous measure of evidence, we adopted a common interpretation (Wagenmakers et al., 2018). According to this interpretation, 1 < BF < 3 is anecdotal (or inconclusive) evidence, 3 < BF < 10 is moderate evidence, 10 < BF < 30 is strong evidence, 30 < BF < 100 is very strong evidence, and BF > 100 is extreme evidence.

For the rating and ERP data, the research questions were addressed with two main analyses using trial‐level data: The first main analysis considered only the data from the first session (pretreatment) and examined whether the combined treatment groups differed from the control group in their responses to spiders versus neutral pictures. In this analysis, the picture category (spider vs. neutral) was modeled as a trial‐varying level‐1 predictor, and the group (VRET/IVET vs. control) was modeled as a trial‐invariant level‐2 predictor. We regressed the dependent variable (i.e., ratings or ERP components) on the fixed effects of picture category, group, and the cross‐level interaction between picture category and group. Intercepts and slopes for picture category were allowed to vary randomly across participants.

The second main analysis included only the two treatment groups and compared both groups in their responses before and after treatment. Thus, this analysis addressed whether the treatment had any effects and whether both groups differed from each other. In this analysis, picture category (spider vs. neutral) and treatment (post vs. pre) were modeled as trial‐varying level‐1 predictors, and group (VRET vs. IVET) was modeled as a trial‐invariant level‐2 predictor. We regressed the dependent variable on the fixed effects of picture category, treatment, and group, and the two‐ and three‐way interactions between them. Intercepts and slopes for treatment, picture category, and their interaction were allowed to vary randomly across participants. To facilitate interpretation of the models, the treatment group was dummy coded as −0.5 (IVET) and 0.5 (VRET) so that the intercept represents the average across treatment groups. The neutral pictures, the pretreatment condition, and the average of both groups (VRET and IVET) were specified as reference levels, such that the intercept is the mean response for neutral pictures before treatment across both groups.

For both main analyses, diagnostics about model assumptions were conducted. For the analyses of the rating data, multicollinearity was not a problem for pleasantness ratings (VIF < 5.4) but arousal ratings (VIF < 42.1). Thus, models for arousal ratings may be biased. Critically, for the analyses of ERP data, multicollinearity was not a problem; for all analyses, VIF < 2.6. Also, diagnostics about linearity and normality did not suggest any problems with the ERP data.

The main analyses and additional analyses are reported in the supplementary material (Wiens & Eklund, 2022). For example, we conducted the same ERP analyses but excluded the target trials (i.e., 20% of trials). Results were similar to those reported below. Also, we analyzed the effects of treatment on positive and negative pictures. Results suggested no notable differences between the control group and the treatment groups. In addition to the Bayesian analyses, we estimated a set of parallel multilevel models using a frequentist approach. Results were comparable; thus, the 95% CIs were very similar to those of the Bayesian analyses and are reported as supplementary material.

## 3 RESULTS

Because the results for the picture rating task provide an important manipulation check, they are reported before the EEG results for the detection task.

### 3.1 Picture ratings

**FIGURE 1:**
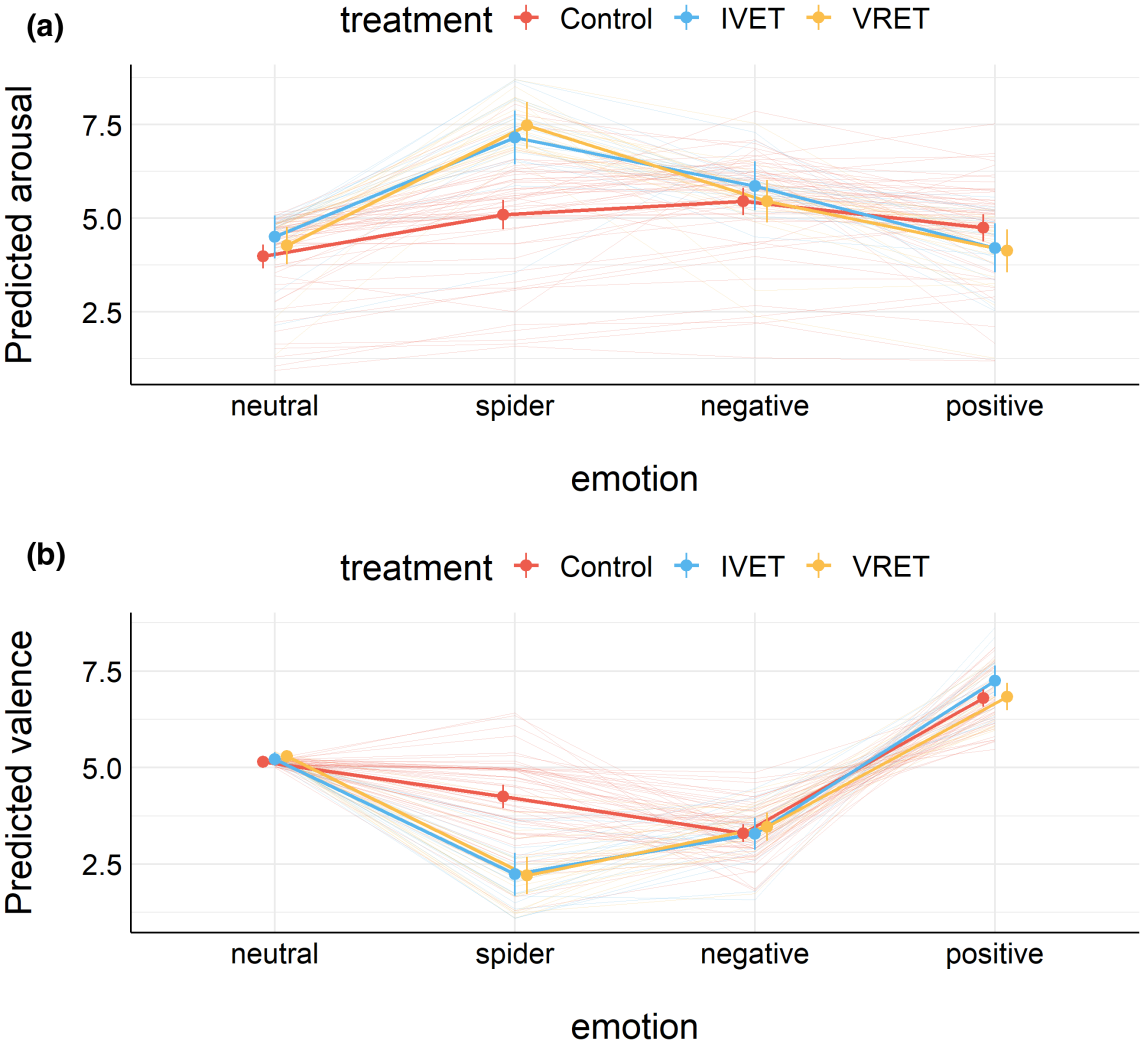
(a) Estimated mean (±95%CI) arousal ratings in Session 1. (b) Estimated mean (±95%CI) pleasantness ratings in Session 1.

**FIGURE 2:**
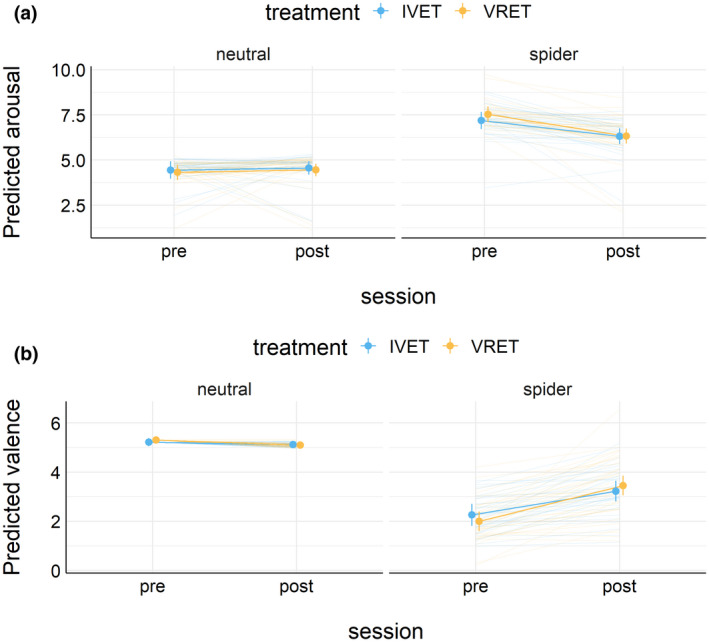
(a) Estimated mean (±95%CI) arousal ratings by the treatment group and session. (b) Estimated mean (±95%CI) pleasantness ratings by the treatment group and session.

At the end of the experiment, participants rated each picture on arousal and pleasantness (on scales between 1 and 9). Figure 1 shows estimated mean ratings (1A: arousal, 1B: pleasantness) for all three groups in Session 1 (before treatment). The figure also shows the estimated means for each individual. This figure suggests that the main differences between the treatment groups and the control group were observed in how subjects rated spiders. Figure 2 shows the estimated mean ratings for spiders and neutral pictures for each treatment group and each session, separately for arousal (Figure 2a) and pleasantness (Figure 2b).

To address the main questions of the present study, subsequent analyses focused on group differences between spiders versus neutral pictures.

### 3.2 Arousal ratings

The first analysis considered only the arousal ratings for all three groups in Session 1 (before treatment). Compared to the control group, the combined treatment groups rated spiders as substantially more arousing than neutral pictures. In support, in a Bayesian ordinal regression, the difference (spider−neutral) in arousal was larger for the combined treatment groups than for the control group; mean difference = 2.21, 95%CI [1.65, 2.81]. A complex model that included the interaction of treatment (patients vs. controls) and picture category (spider vs. neutral), as well as lower‐order effects, received extreme support compared to a simple model that included only picture category, *BF* = 6.3 * 10e+8.

The second analysis compared the two treatment groups before and after treatment. Results showed that arousal ratings to spiders (vs. neutral) were lower after treatment relative to before treatment, and this effect did not vary by treatment group. In support, a Bayesian ordinal regression showed that higher arousal ratings to spiders (vs. neutral) decreased with treatment (post vs. pre); mean difference = −1.27, 95%CI [−1.80, −0.71]. A model that included the interaction of treatment (post vs. pre) and picture category (spider vs. neutral), as well as lower‐order effects, received very strong support compared to a simple model that included only picture category, *BF* = 47. Results suggested that the interaction of treatment and picture category did not vary by treatment group: Mean difference = −0.47, 95%CI [−1.54, 0.65]. The simpler model without the interaction of treatment (post vs. pre), picture category (spider vs. neutral), and treatment group (VRET vs. IVET) received extreme support compared to the complex model, *BF* = 3.2 * 10e+6.

### 3.3 Pleasantness ratings

The first analysis showed that the difference (spider−neutral) in pleasantness was larger for the combined treatment groups than for the control group; mean difference = −2.71, 95%CI [−3.38, −2.06]. Thus, compared to the control group, the combined treatment groups rated spiders as less pleasant than neutral pictures. A complex model that included the interaction of treatment (patients vs. controls) and picture category (spider vs. neutral), as well as lower‐order effects, received extreme support compared to a simple model that included only picture category, *BF* = 7.2 * 10e+8.

The second analysis showed that the pleasantness ratings for spiders (vs. neutral) increased with treatment (post vs. pre); mean difference = 1.73, 95%CI [1.21, 2.27]. A model that included the interaction of treatment (post vs. pre) and picture category (spider vs. neutral), as well as lower‐order effects, received extreme support compared to a simple model that included only picture category, *BF >*1.6 * 10e+22. Results suggested that the interaction of treatment and picture category did not vary by treatment group: Mean difference = 0.50, 95%CI [−0.60, 1.58]. The simpler model without the interaction of treatment (post vs. pre), picture category (spider vs. neutral), and treatment group (VRET vs. IVET) received extreme support compared to the complex model, *BF* = 4395.

### 3.4 EEG

**FIGURE 3:**
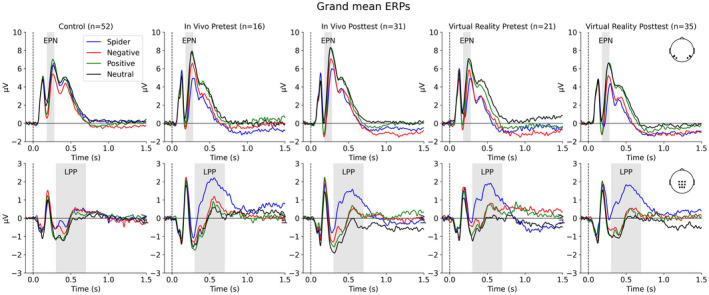
Grand mean ERPs for EPN‐ and LPP‐relevant electrodes.

**FIGURE 4:**
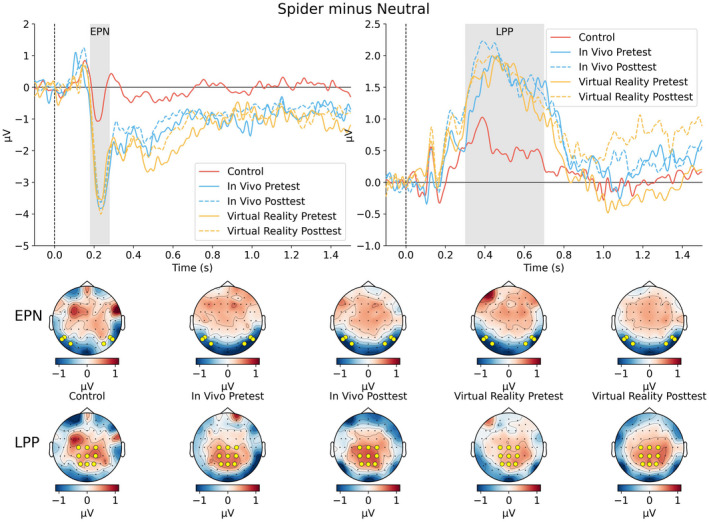
Mean difference waves and topographies.

Figures 3 and 4 illustrate the quality of the EEG data and support the choice of intervals for EPN and LPP. The top row in Figure 3 shows grand mean ERPs across EPN‐relevant electrodes, and the bottom row shows grand mean ERPs across LPP‐relevant electrodes for the control group, IVET group (pre and post), and VRET group (pre and post). The top row in Figure 4 shows the mean difference waves of spider minus neutral for the different groups, and the bottom row shows topographies of the difference waves for the chosen intervals (i.e., EPN and LPP).

### 3.5 EPN

**FIGURE 5:**
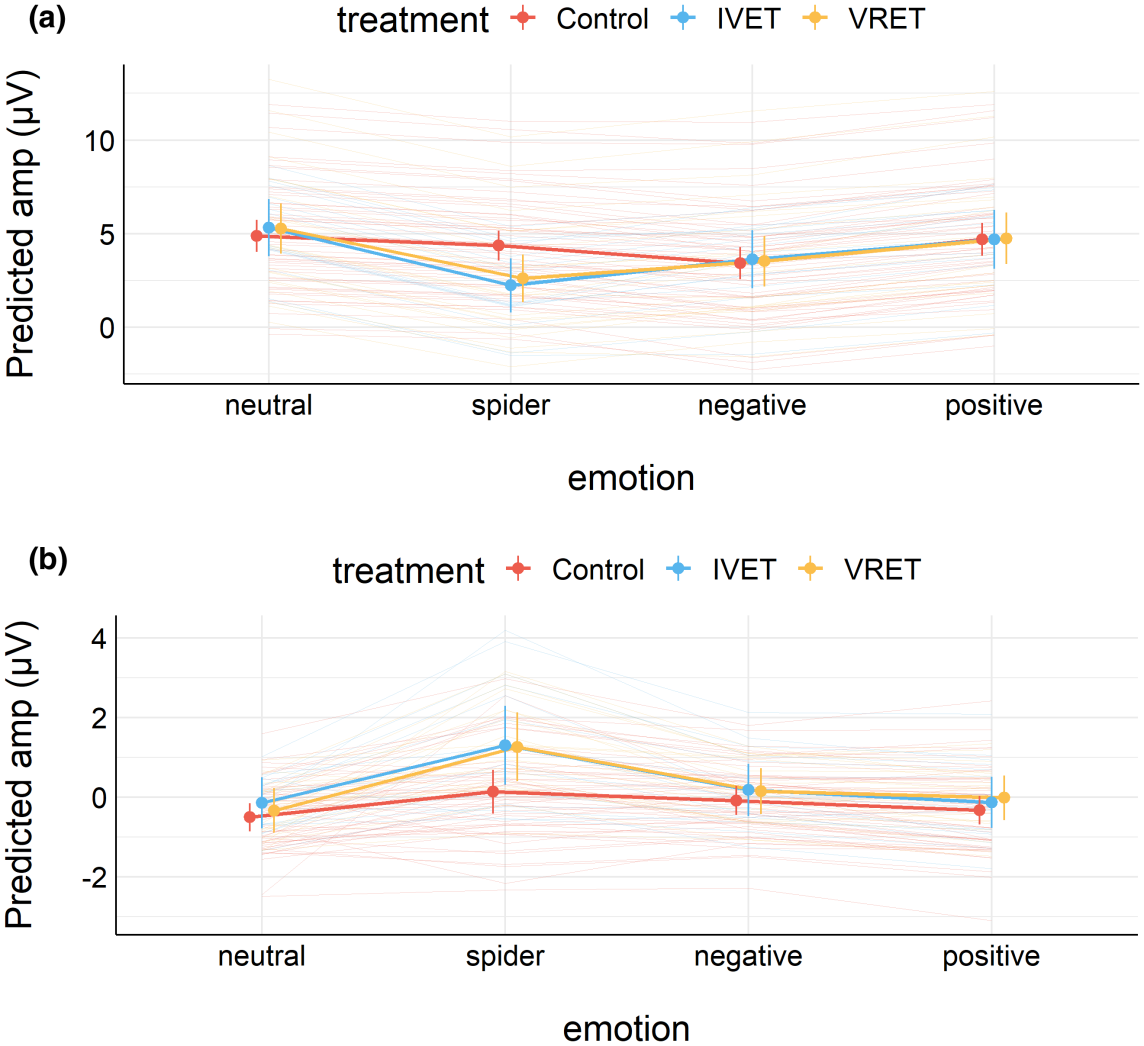
(a) Estimated EPN‐relevant mean (±95%CI) amplitudes in Session 1. (b) Estimated LPP‐relevant mean (±95%CI) amplitudes in Session 1.

**FIGURE 6:**
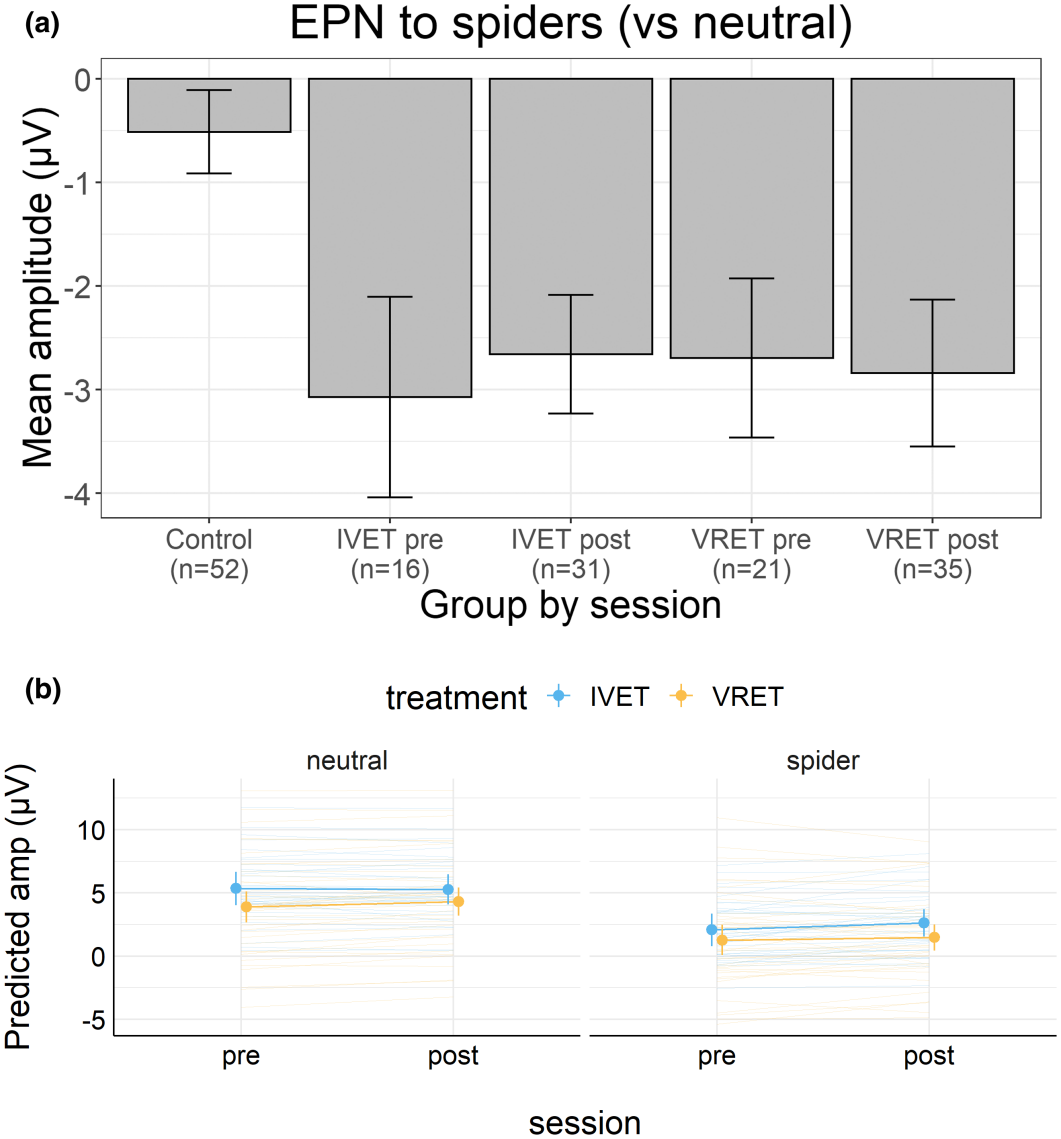
(a) Observed EPN mean (±95%CI) amplitudes by the treatment group and session. (b) Estimated EPN‐relevant mean (±95%CI) amplitudes by the treatment group and session.

Figure 5a shows the estimated EPN‐relevant mean amplitudes for the four picture categories in Session 1 (before treatment), separately for the three groups. The figure also shows the estimated means for each individual. Figure 6a shows the observed mean EPN (i.e., spiders minus neutral) for each treatment group and each session (and for the control group). Figure 6b shows the estimated EPN‐relevant mean amplitudes.

Similar to the analyses of the rating data, the EPN‐relevant data were analyzed in two ways. The first analysis showed that the combined treatment groups versus the control group showed less positive amplitudes to spiders than neutral pictures. Because relative negativity in amplitudes between spiders and neutral is characteristic of the EPN, these results show that the combined treatment groups had a larger EPN than did the control group. In support, a Bayesian analysis of the combined treatment groups versus the control group in terms of the difference between spider and neutral pictures showed a negative amplitude difference; mean difference = −2.32, 95%CI [−2.99, −1.65]. A complex model that included the interaction of treatment (patients vs. controls) and picture category (spider vs. neutral), as well as lower‐order effects, received extreme support compared to a simple model that included only picture category, *BF* = 1 * 10e+7.

The second analysis suggested that the EPN did not vary with treatment or with treatment groups. In support, a Bayesian analysis of the mean amplitude difference between spider and neutral pictures before and after treatment showed that the relative negativity to spiders was unaffected by treatment; mean difference = 0.12, 95%CI [−0.43, 0.67]. This contrast between picture category and treatment did not interact with the treatment group; mean difference = −0.79, 95%CI [−1.89, 0.33]. In a model comparison, a simple model that included only picture category (spider vs. neutral) received extreme support compared to a complex model that included the interaction of picture category and treatment (*BF* = 177) and received extreme support compared to a more complex model that included the three‐way interaction with the treatment group (*BF* = 1.3 * 10e+5).

### 3.6 LPP

**FIGURE 7:**
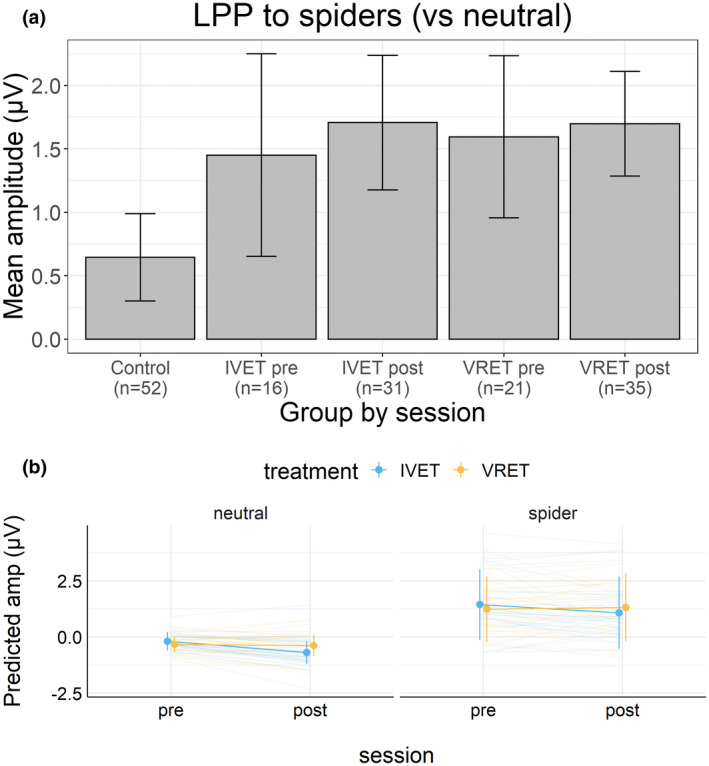
(a) Observed LPP mean (±95%CI) amplitudes by the treatment group and session. (b) Estimated EPN‐relevant mean (±95%CI) amplitudes by the treatment group and session.

Figure 5b shows the estimated LPP‐relevant mean amplitudes for the four picture categories, separately for the three groups. Figure 7a shows the observed mean LPP (i.e., spiders minus neutral) for each treatment group and each session (and for the control group). Figure 7b shows the estimated LPP‐relevant mean amplitudes.

The first analysis showed that compared to the control group, the combined treatment groups showed more positive amplitudes to spiders than neutral pictures. Because the relative positivity in amplitudes between spiders and neutral is characteristic of the LPP, these results show that the combined treatment groups had larger LPP to spiders than the control group. In support, a Bayesian analysis of the combined treatment groups versus the control group in terms of the difference between spider and neutral pictures showed a positive amplitude difference; mean difference = 0.86, 95%CI [0.30, 1.41]. However, the model comparison could not resolve (*BF* = 1.9) between a complex model that included the interaction with treatment (patients vs. controls), as well as lower‐order effects, and a simple model that included only picture category (spider vs. neutral).

The second analysis suggested that LPP did not vary with treatment or with treatment groups. In support, a Bayesian analysis of the mean amplitude difference between spider and neutral pictures before and after treatment showed that the relative positivity to spiders was unaffected by treatment; mean difference = 0.08, 95%CI [−0.37, 0.53]. This contrast between picture category and treatment did not interact with the treatment group; mean difference = 0.03, 95%CI [−0.88, 0.91]. In a model comparison, a simple model that included only picture category (spider vs. neutral) received strong support when compared to a complex model that included the interaction of picture category and treatment (*BF* = 13,723) and received extreme support when compared to a more complex model that included the three‐way interaction with treatment group (*BF* = 9 * 10e+7).

### 3.7 Detection task performance

In the detection task, subjects made very few false alarms overall in detecting the blinking of the fixation cross; mean number of false alarms = 1.4 per session. Therefore, these trials were ignored. Hits were target trials in which subjects detected the blinking of the fixation cross and did not respond in less than 200 ms after the blink onset. Mean hit rate per session was 78.5%. Because there were only 10 target trials per picture category, hit rates were compared between spiders (10 trials) and nonspiders (30 trials). The analysis of the hit rates before treatment showed that relative to the control group, the combined treatment groups tended to have lower hit rates to spiders (vs. nonspiders), mean difference = −6.42, 95%CI [−12.97, −0.13]). The analysis of the hit rates for the two treatment groups over sessions showed that the difference in hit rates between spiders and nonspiders before treatment (mean difference = −5.79) was lower after treatment (mean change = 7.85, 95%CI [1.46, 14.23]). Results did not suggest that this effect varied with treatment. Analyses of reaction times to hits did not suggest any effects (see supplement).

### 3.8 Task experience

After the task, subjects answered questions about their experience of the task (on scales ranging from 1 to 9). Detailed analyses are reported as supplementary material. In general, participants rated that they did focus on the fixation cross (*M* ≈ 8 where 1 = never and 9 = always), that the flashing cross was somewhat difficult to see (*M* ≈ 4 where 1 = difficult and 9 = easy), and the task was neither easy nor difficult (*M* ≈ 5). In terms of group differences, the combined treatment groups (vs. control group) reported being more distracted by spiders than nonspiders in the first session (*M* diff ≈ 3). After treatment (vs. before treatment), the combined treatment groups were less distracted by spiders (vs. nonspiders), *M* diff ≈ − 1.5, and rated the task as easier, *M* diff ≈ 0.8.

## 4 DISCUSSION

The main results were that before treatment (IVET and VRET), participants with spider phobia responded specifically to spiders: Compared to neutral pictures and the control group, patients rated spiders as more arousing and less pleasant, and showed larger EPN and LPP suggesting they experienced stronger affective responses when viewing spiders. One week after treatment, patients rated spiders as less arousing and less pleasant, whereas EPN and LPP were unaffected, highlighting an interesting dissociation between subjective and neural responses. Finally, these effects did not differ by treatment group (IVET and VRET), indicating that in vivo and virtual reality treatment were equally as effective in reducing affective responses to spiders.

In the present study, EPN and LPP were observed during a simple detection task, consistent with previous reports of EPN and LPP to task‐irrelevant emotional pictures at fixation (Norberg et al., 2010; Norberg & Wiens, 2013; Nordström & Wiens, 2012; Sand & Wiens, 2011; Schupp et al., 2007; Wiens et al., 2012; Wiens & Syrjänen, 2013). When compared to control participants, participants with spider phobia reported that they were more distracted by spider pictures than neutral pictures before treatment. After treatment, participants with spider phobia reported that they were less distracted by spiders. Performance (hit rates) on the detection task showed a similar pattern. These findings confirm that pictures were processed even though they were task irrelevant.

Compared to participants in the control group, participants in the combined treatment groups (IVET and VRET) responded strongly to spiders (vs. neutral pictures) before treatment. With regard to ratings of arousal and pleasantness, the difference (spider−neutral) was larger for the combined treatment groups than for the control group. Similar results were obtained for EPN (Figures 5a and 6a) and LPP (Figures 5b and 7a). These findings provide manipulation checks for ratings, EPN, and LPP. These findings are consistent with motivational theories of emotion (Bradley, 2009; Lang et al., 1997; Lang & Bradley, 2010) and support the conclusion that for people with spider phobia, spiders are particularly motivating and capture attention.

One week after treatment, participants with spider phobia rated spiders (vs. neutral pictures) as substantially less arousing and less unpleasant than before treatment, as shown in Figure 1a,b. For arousal ratings, results from Bayesian analyses suggested that this effect did not interact with the treatment group. For pleasantness, results from Bayesian analyses suggested that this effect interacted with the treatment group; however, no notable differences were apparent and the 95%CI of the three‐way interaction overlapped with zero. As shown in Figures 6b and 7b, results suggested that EPN and LPP to spiders (vs. neutral pictures) were unaffected by treatment. Bayesian analyses suggested that the data provided moderate to extreme evidence in favor of a simple model that included only picture category (spider vs. neutral) compared to more complex models.

Taken together, the present results suggest that IVET and VRET showed comparable responses in terms of ratings, EPN, and LPP. Also, after treatment, spiders were rated as less arousing and less unpleasant. These results may not be surprising because both treatment modalities showed strong effects on self‐reported fear and behavioral avoidance (Miloff et al., 2019). Critically, the present results suggest that treatment did not affect EPN and LPP to spiders (vs. neutral).

One possible explanation is that the present finding of no changes in EPN and LPP as a result of therapy is a *false negative*. That is, although there is a true effect (i.e., EPN and LPP decrease), the present data failed to detect it. Importantly, the statistical inferences in the present study do not rely on null hypothesis significance testing (Wasserstein & Lazar, 2016; Wiens & Nilsson, 2017). In numerous studies, it is often concluded that there is no effect because the test was not significant (*p >* .05). However, this conclusion is statistically invalid (particularly so if there is no a priori power analysis) (Dienes, 2008; Makin & Orban de Xivry, 2019; Wasserstein & Lazar, 2016). In contrast, Bayesian hypothesis testing is a valid procedure to compare different models and to provide evidence for or against a particular model (Dienes, 2016; Wagenmakers et al., 2016; Wiens & Nilsson, 2017). However, despite this advantage of Bayesian analyses, they are bound by the data because the truth is unknown. Accordingly, we can conclude only that the present data suggest that EPN and LPP are unaffected by treatment, but the evidence may shift as more studies with new data are added.

Another possible explanation consistent with the data is that there is no true effect (i.e., EPN and LPP are unaffected by therapy). In fact, changes in ratings may not be accompanied necessarily by changes in EPN and LPP, and vice versa (Langeslag & van Strien, 2018; Paul, Kathmann, & Riesel, 2016; Paul, Simon, et al., 2016). For example, when nonfearful participants viewed spiders and snakes and were asked to up‐regulate or down‐regulate their emotional responses by reappraisal, LPP was larger during up‐ than down‐regulation whereas EPN was apparently unaffected (Langeslag & van Strien, 2018). Thus, effects on ratings may actually differ from those on EPN and LPP.

A possible reason for this discrepancy is that ratings and ERPs were recorded during different tasks. However, it is common practice to record EEG separately from emotion ratings (Hajcak et al., 2011). In previous treatment studies, EEG was recorded during passive picture viewing, and emotion ratings were recorded during a subsequent rating task (Leutgeb et al., 2009, 2012). In the present study, EPN and LPP were recorded during a detection task, and ratings were recorded during a subsequent picture rating task. Although picture duration was identical in both tasks (1500 ms), the detection task required participants to attend the fixation cross rather than the pictures, whereas the rating task required participants to focus on the pictures. The main goal of the detection task (rather than passive viewing) was to reduce the risk of potentially confounding effects of attention shifts on EPN and LPP. Another advantage of the detection task was that because participants had to perform an active task (rather than passive viewing), this task likely prevented confounding effects from differences in emotion regulation (Hajcak et al., 2010).

At face value, a discrepancy between effects on ratings and EPN and LPP is inconsistent with motivational theories of emotion (Bradley, 2009; Lang et al., 1997; Lang & Bradley, 2010). According to these theories, if pictures are rated as less negative after than before treatment, their motivational strength and motivated attention should be lower, and EPN and LPP should decrease accordingly. However, such a dissociation between measures of emotion does not challenge motivational theories of emotion per se. These theories emphasize that self‐report, overt behavior, and physiology are valid measures of emotion. However, the theories are not explicit about the direct link between different measures and the theoretical construct of fear; thus, all measures do not need to show similar results (Miller & Kozak, 1993). Accordingly, if EPN and LPP on the one hand and perceived valence and arousal, on the contrary, show different results, this dissociation suggests that these measures represent different functional processes. That is, whereas monitoring the environment for emotionally relevant stimuli may be the main process involved in generating the EPN and LPP, this process is likely to play less of a role during picture rating.

A lack of change in EPN and LPP after exposure therapy is also interesting from the perspective of inhibitory learning. Inhibitory learning is a relatively new theory on how extinction learning occurs after exposure therapy (Craske et al., 2014). Whereas the well‐known theory of habituation stipulates that treatment weakens or replaces preexisting links between stimuli and responses held in memory, inhibitory learning stipulates that these structures remain intact but new competing learning is acquired that inhibits prior associations with danger (Weisman & Rodebaugh, 2018). In this study, despite large changes in picture ratings after treatment, as well as improvements in behavioral avoidance and self‐reported fear (Miloff et al., 2019), EPN and LPP were unaffected. Because this aspect of the emotional response to spiders remained similar before and after treatment, this lack of change is consistent with inhibitory learning theory. However, as in previous studies (Leutgeb et al., 2009, 2012), data in the present study were recorded only 1 week after treatment. Therefore, it is unresolved whether EPN and LPP would remain unaffected beyond 1 week.

Because previous studies as well as the present study measured ERPs and picture ratings during separate tasks, it is unresolved whether EPN and LPP might be affected by treatment when they are recorded during a picture rating task, which is preferable to passive viewing (Bernhardsson et al., 2016; Leutgeb et al., 2009, 2012). If EPN and LPP are recorded during a picture rating task (or passive viewing) before and after treatment, then it is important to record eye movements as well (Bernhardsson et al., 2016) to examine potentially confounding effects of differences in gaze on EPN and LPP. Future studies should also measure long‐term treatment effects to determine whether EPN and LPP may be unaffected in the long run, consistent with inhibitory learning theory (Craske et al., 2014).

To conclude, the present study found that although exposure therapy (IVET and VRET) reduced ratings of arousal and unpleasantness to spiders 1 week after therapy, EPN and LPP were unaffected. Effects did not vary by treatment modality (IVET and VRET). Because EPN and LPP were recorded while pictures were task irrelevant, the absence of treatment effects on EPN and LPP suggests that the initial stages of motivated attention are unaffected by treatment.

## AUTHOR CONTRIBUTIONS

**Stefan Wiens:** Conceptualization; data curation; formal analysis; funding acquisition; investigation; methodology; project administration; resources; software; supervision; validation; visualization; writing – original draft; writing – review and editing. **Rasmus Eklund:** Conceptualization; data curation; formal analysis; investigation; methodology; project administration; resources; software; validation; visualization; writing – original draft; writing – review and editing. **Malina Szychowska:** Conceptualization; data curation; investigation; methodology; project administration; writing – review and editing. **Alexander Miloff:** Conceptualization; data curation; formal analysis; methodology; project administration; writing – review and editing. **Danielle Cosme:** Formal analysis; software; visualization; writing – review and editing. **Stephen Pierzchajlo:** Formal analysis; software; visualization; writing – review and editing. **Per Carlbring:** Conceptualization; funding acquisition; project administration; resources; supervision; writing – review and editing.
